# Indian Plastic Surgery Teams Lead with the World's Highest Number of Hand Transplants

**DOI:** 10.1055/s-0044-1792155

**Published:** 2024-11-15

**Authors:** Dinesh Kadam

**Affiliations:** 1 Department of Plastic and Reconstructive Surgery, A J Institute of Medical Sciences and Research Centre, Mangalore, Karnataka, India

It is a matter of pride and privilege for us that Indian plastic surgeons quietly lead the world in the highest number of hand transplants. Completing an impressive 73 upper extremity transplants as of September 2024, India has surpassed all other countries ( Fig. 1 ). In addition, India has performed the highest number of double hand transplants, with a total of 33 patients, which is a testament to its high resource efficiency. Gaining experience with successive transplants, quick collaboration, and comprehensive training sessions, our plastic surgeons have performed transplants successfully in a relatively short time.

**Fig. 1:**
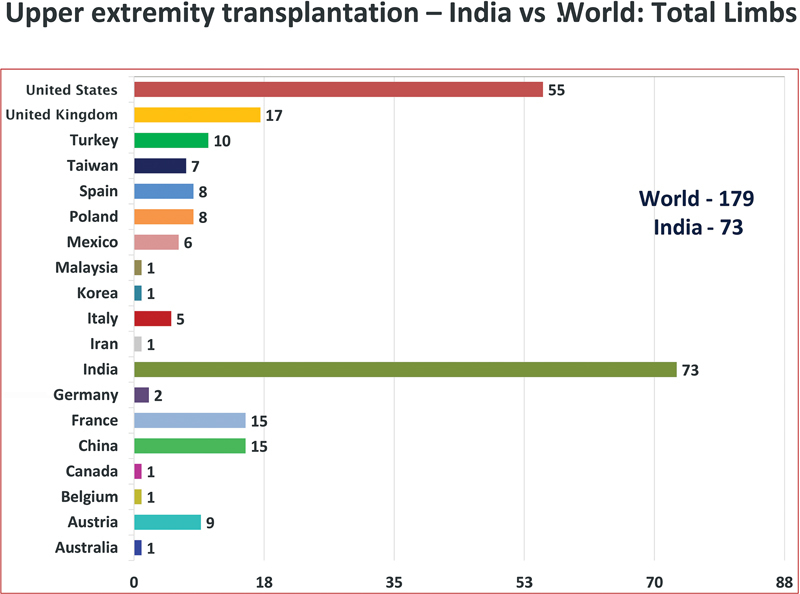
Number of hand transplants in the world. (Image Courtesy: Dr Mohit Sharma and Dr Selva SeethaRaman).

India was the 15th country to perform hand transplants in 2015, relatively later than the world's first successful hand transplant in 1998. With several successful transplants in different institutions nationwide, the momentum in India is high, exceeding that of many countries. I am pleased to present preliminary data gathered through personal communications and voluntary reporting of institutions ( Table 1 ). India's achievements in hand transplantation need global attention as the country is poised to make even greater strides in this area.

**Table 1:** Hand (upper extremity) transplant data of India a

	Hospital	Place	Year	Patients	Hands	Lead plastic surgeons
1	Amrita Hospitals	Kochi	2015	15	30	Dr. Subramania Iyar et al Dr. Mohit Sharma et al
2		Faridabad-NCR	2023	02	03	
3	Gleneagles Hospitals	Mumbai	2020	12	22	Dr. Nilesh Satbhai et al
4		Chennai	2021	03	05	Dr. Selva SeethaRaman et al	
5	JIPMER	Pondicherry	2017	03	06	Dr. Dinesh Kumar/Dr. Friji/Dr. Mohapatra/Dr. Ravi Chittoria
6	Stanley Medical College	Chennai	2017	01	02	Dr. Ramadevi/Dr. Jeyakumar P. et al
7	Sir Ganga Ram Hospital	Delhi	2024	01	02	Dr. Mangal Mahesh/Dr. Anubhav Gupta et al
8	IPGMER-SSKM Hospital	Kolkata		01	02	Dr. Arindam Sarkar et al
9	KEM Hospital	Mumbai	2021	01	01	Dr. Vinita Puri/Dr. N. Venkateshwaran et al
Total				39	73	Remarks: Rejection salvages: 7 Explantations: 2	

a Data as of September 21, 2024 (personal communication).

## Hand Transplants in India from 2015

**Fig. 2:**
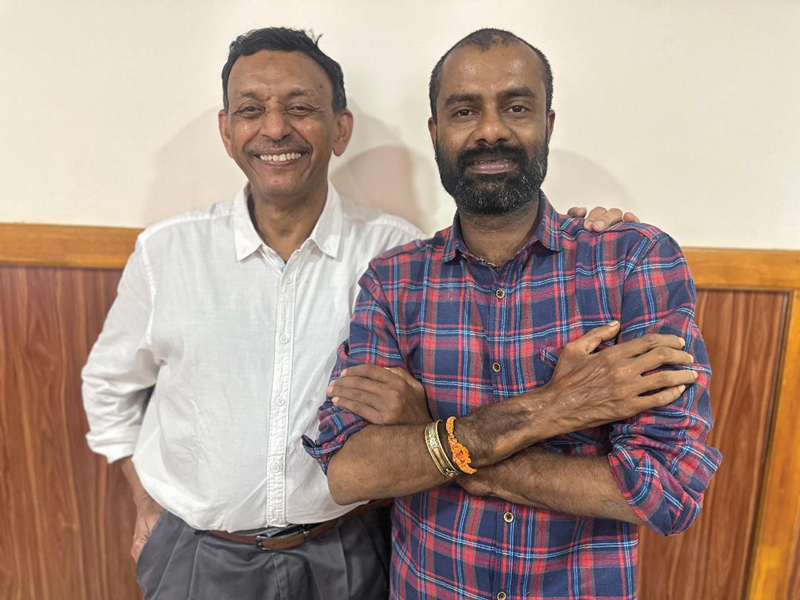
Prof. Subramania Iyer with the first hand transplant recipient after 9 years.

In 2015, Amrita Hospital in Kochi performed the historic first double hand transplant in the Indian subcontinent under the leadership of Dr. Subramania Iyer and Mohit Sharma. 1 Nine years since, the recipient, living a blissful family life and taking the role of a transplant coordinator at Amrita, remains a brand ambassador of hand transplants in India ( Fig. 2 ). With his considerable clinical experience, leading the Amrita Institutions with the highest hand transplants for any institution in the world, Dr. Iyer remains a core support group and trainer for hand transplants in India. Dr. Mohit Sharma has expanded hand transplant services to North India in the new Amrita Hospital, NCR Delhi. The Amrita team is also credited with the first Asian bilateral transplant at the supracondylar level. 2

In 2017, Jawaharlal Institute of Postgraduate Medical Education and Research (JIPMER), Pondicherry, became the second institution to perform hand transplants under Dr. Dinesh Kumar and his team. 3 JIPMER is the first government institution to offer hand transplant services and also the first to conduct cross-gender transplants from female to male. In the same year, Stanley Medical College, Chennai, performed a double hand transplant under the leadership of Dr. Rama Devi and Dr. Jeykumar. In fact, Prof R.Krishnamoorthy initiated the process by conducting First Indian Symposium on Hand Transplant symposium at Stanley Medical College in 2010 inviting Dr Vijay Gorantla of USA.

**Fig. 3:**
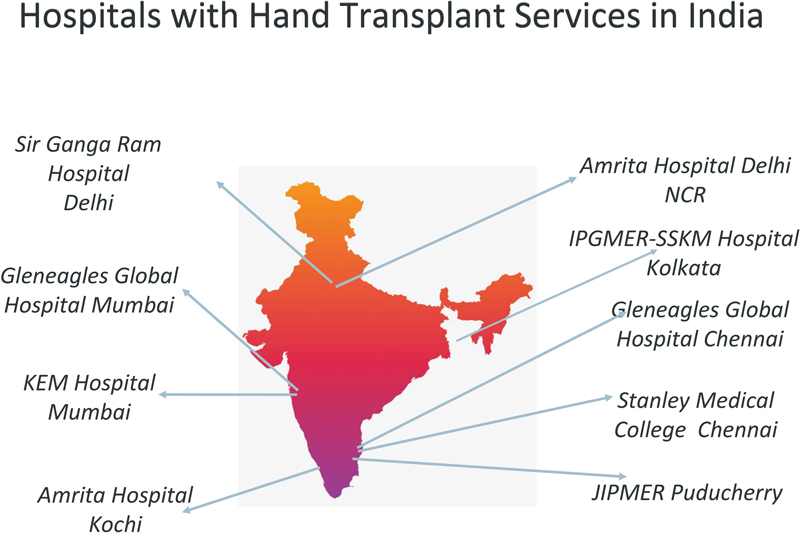
Hand transplant centers in India.

A remarkable feat under Dr. Nilesh Satbhai has seen Mumbai performing over 22 upper extremities in a short span of a few years from 2020. At Gleneagles Hospital Mumbai, Dr. Nilesh is the first to perform a partial hand transplant, a hand transplant in a congenital anomaly, and is also credited with the longest distance organ retravel transplant. Dr. Selva SeethaRaman, who has been part of many hand transplants in the country, has successfully performed five hand transplants at Gleneagles Hospital Chennai. Among the nine centers that have performed hand transplants are IPGMER, Kolkata, KEM hospital, Mumbai, and Sir Ganga Ram Hospital, Delhi ( Fig. 3 ).

## Highest Bilateral Hand Transplants in the World

**Fig. 4:**
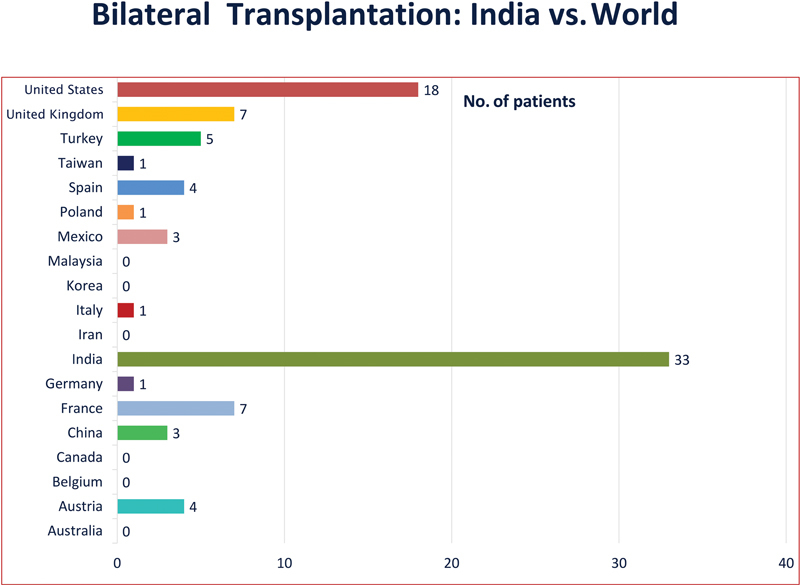
Bilateral hand transplants in the world. (Image Courtesy: Dr Mohit Sharma and Dr Selva SeethaRaman).

Despite technical and logistical enormity, India has rarely lost an opportunity to perform a double hand transplant ( Fig. 4 ). With the exception of a few specific indications, such as patients already under the immunosuppression or congenital absence, all have been double hand or attempted double hand transplants. It is much more logical to restore the hands of bilateral amputees to rehabilitate functionally, socially, and psychologically. It fulfils several functional, ethical, and logistical criteria where the young bilateral hand amputees are the most deserving on the waiting list. Adding quality to life is the most reasonable trade-off for the potential adverse effects of lifelong immunosuppression, as well as the effort and cost of the entire exercise. This also underscores India's united, well-coordinated efforts, where surgeons from different parts of the country join the team for a long haul for calls at the 11th hour.

## Cadaveric Workshops and Rehearsal Sessions

Hand transplant is distinctly different and much beyond the technical extension of major replantation. A reconstructive microsurgeon must understand the basic principles of vascularized composite allografts (VCA) and the distinction between solid organ transplants. Good knowledge of immunosuppression regimens, medical management, and complications is essential. Performing a hand transplant requires massive teamwork. Dr. Selva SeethaRaman, who is part of many teams, elaborates that, for a bilateral hand transplant, ideally, there should be 10 plastic surgeons to make 5 teams of 2 surgeons each. Two teams are for harvesting, two for recipient preparation, and one to rotate among them. Besides teams of anesthetists and intensivists, four orthopaedicians are required for bilateral skeletal fixation. It is absolutely essential that all of them are clear about their roles and the sequence of the procedure. A surgical drill to rehearse the entire sequence of limb retrieval to the transplantation is essential, urges Dr. Vinita Puri, who has taken the lead in convening cadaver training programs at KEM, Mumbai. Since 2016, over six workshops have been held at the Cadaver Laboratory at KEM, Mumbai, Nair Hospital, Mumbai, and AIIMS, Delhi. These training courses also elaborate on preparing the institution for transplants with licensing and familiarity with government guidelines. Such workshops have been instrumental in the success of the hand transplant program in India.

## India Emerging Despite Challenges

Even after 25 years since the first successful hand transplant, the number of hand transplants worldwide is few, precious, and makes headlines. The group at CM Kleinert Institute with the University of Louisville, United States, is credited with the first successful, longest-surviving hand transplant. They pioneered the research from preclinical trials, forming protocols to clinical success. Despite the robust clinical experience, the program was discontinued after 10 transplants due to various limitations, including funding and insurance, before being restarted under the collaboration. 4 Transplants performed in China resulted in 58% graft loss following initial success due to noncompliance or lack of access to immunosuppression. 5 South Korea began its program only in 2021, after formulating legislation for transplants. 6 The cost remains a major limiting factor, beginning with expensive surgery followed by lifelong immunosuppression. The economic cost–utility analysis of hand transplantation in the United States showed an overall lifetime cost of $530,000, including 40 years of immunosuppression.

The cost is similar in Indian private hospitals, with Rs. 15 to 30 lakhs for a bilateral hand transplant procedure; however, many are supported by philanthropic funding. Government hospitals absorb most of the expenses and provide free immunosuppressive drugs. Dr. Jeykumar states that the government of Tamil Nadu provides free immunosuppression drugs even for private hospital patients. The overall monthly cost is much lower in India, approximately Rs. 15,000 per month, a relatively affordable out-of-pocket expense for the recipient. This makes long-term sustainability a reality, notwithstanding any complications or rejections that may incur additional costs for institutions, surgeons, and recipients.

## Hand Transplant Registry

To implement the transplant program activities, the government of India has established the National Organ and Tissue Transplant Organization (NOTTO), an apex organization at the national level. Similar organizations at the regional and state levels (ROTTO/SOTTO) provide an efficient and organized system of organ procurement and distribution in the country and to maintain a national registry of donors and recipients of organs and tissues.

Until this year, hand transplants were not part of the NOTTO registry. In July 2024, NOTTO notified that hand transplants should be registered in the transplant registry and categorized under bone tissue. 7 This will ensure more comprehensive data collection, structured outcomes reporting, and greater organ distribution efficiency between centers.

As per the 2023 NOTTO data, India ranks third in the world in organ transplantation and second in corneal transplantation. 8 With more than 1,000 deceased organ donations (1,099) for the first time, India has breached its own record, reporting the highest number of deceased organ donations. Despite the milestone achievements, organ donation in India remains low, with less than one donor per million population per year. One of the key challenges is poor identification and certification of brain stem death (BSD) patients despite many potential and willing donors available. As per the provisions of the Transplantation of Human Organs and Tissues Act, it is required to identify each potential BSD patient in the intensive care unit and mandatorily inquire about the patient's pledge for organ donation and make the family aware of the possible opportunity to donate organs before the heart stops. However, in reality, the process of certification, organ harvest, and transport is an elaborate process that requires the coordination of different teams and logistical and financial support.

Informing relatives of cadaver donors that the limbs will be replaced with prostheses after the hand retrieval and not left mutilated is essential for hand transplantation.

## Our Responsibility as Global Leaders

India's potential for further hand transplantation is enormous. With around 1,099 deceased organ donors in 2023, potentially 2,000 hands, and an estimated 110 upper extremity amputations per million annually, there is room for scaling up. However, a substantial financial commitment is required to support both surgeries and long-term care for recipients. Economic challenges, lack of sufficient transplant coordinators, and logistical hurdles continue to limit the full potential of the program despite India's leadership in hand transplants. As we expand hand transplant programs nationwide, we are responsible for reporting comprehensive outcome data; the global community will be watching closely, recognizing India as a leader in this challenging field.
